# Transcriptome and Proteome Changes in Silk Gland of Silkworm (*Bombyx mori*) Following Oral Feeding of Sodium Fluoride

**DOI:** 10.3390/biology15201813

**Published:** 2026-10-10

**Authors:** Shuo Li, Xi Zheng, Ting Yang, Shuiyan Yang, Hao Tang, Chao Li, Guannan Li, Benhua Xiong

**Affiliations:** 1 Chongqing Sericulture Science and Technology Research Institute, Chongqing 400700, China; stoneleafli@163.com (S.L.); ymt0202@163.com (T.Y.); sxlqzzyy2008@163.com (S.Y.); htang1010@163.com (H.T.); lichao19911991@gmail.com (C.L.); 2 State Key Laboratory of Resource Insects, College of Sericulture, Textile and Biomass Sciences, Southwest University, Chongqing 400716, China; ccstone2019@email.swu.edu.cn (X.Z.); lgn20220504@swu.edu.cn (G.L.)

**Keywords:** fluoride exposure, silkworm, silk gland, transcriptome, proteome

## Abstract

Fluoride contamination is a long-standing persistent environmental issue, and the silkworm (*Bombyx mori*) has been recognized as superior as a model organism for toxicological studies. When exposed to fluoride, silkworms can accumulate twice the fluoride content of ingested mulberry leaves. Growth and development are severely slowed. The mechanisms of fluoride toxicity have been studied to a small but unclear extent. In this study, we performed transcriptome and proteome sequencing analysis of the silk glands of silkworms under fluoride exposure and regular feeding. We identified 67 differentially expressed genes (DEGs) and 671 differentially expressed proteins (DEPs). By GO and KEGG analysis, we identified three critical pathways, Ribosome biogenesis in eukaryotes, Protein export, and Protein processing in endoplasmic reticulum. In these three pathways, *BmSPCS2*, *BmSPCS3*, and *BmHsp20.8* were significantly down-regulated and *BmHEATR1* was significantly up-regulated. We found that fluoride exposure severely affected protein translation and folding. Our findings provide speculative insights into the molecular mechanism of fluoride toxicity in silkworm silk glands at the transcriptomic and proteomic levels. It allows further expansion of the study on the toxicity mechanism of fluoride in silkworms. It also provides essential gene/protein sequence information for insect toxicology studies.

## Simple Summary

Fluoride contamination is a widespread, persistent environmental problem, and silkworms are excellent model organisms for toxicological research. Silkworms accumulate high levels of fluoride after eating contaminated mulberry leaves, resulting in severe growth retardation. Nevertheless, the molecular mechanism of fluoride toxicity in silkworms remains poorly understood. In this study, we performed transcriptomic and proteomic sequencing on silk glands from fluoride-exposed and normally fed silkworms. We identified 67 differentially expressed genes and 671 differentially expressed proteins. Our analysis revealed three core biological pathways related to protein biosynthesis and folding, with several key genes showing obvious expression changes. We conclude that fluoride exposure disrupts protein translation and folding in silkworm silk glands. These findings clarify the toxic effect of fluoride on silkworm silk glands and provide valuable molecular resources for insect toxicology research.

## 1. Introduction

Fluorine is widely distributed in nature and comes from diverse sources, mainly fluorapatite formed during crustal magma melting. Fluorine is a trace element that is critical for maintaining normal human physiological functions [1], but it is also a cumulative toxicant. If the organism ingests it for a long time, it will accumulate in the organism. Excessive levels of fluorine in the body affect growth and development [2], digestion [3], respiration [4], reproduction [5,6], nervous system [7]. Fluoride pollution has also caused substantial adverse impacts on the sericulture industry in sericultural regions. In the southwestern sericultural areas of China, cases of total cocoon crop failure induced by fluoride pollution have been documented, resulting in severe economic losses for silkworm farmers [8].

Studies have shown that fluoride is absorbed in the midgut by facilitated diffusion. Fluoride is transported across biological membranes primarily via a pH-dependent, carrier-mediated pathway and acts as an ionophore in vivo that disrupts calcium homeostasis by chelating endogenous calcium ions [9,10]. Mitochondria are the key intracellular fluoride targets [11]. When fluoride enters cells, it disrupts mitochondria, promoting the release of cytochrome C and inducing apoptosis [12]. Fluoride ions inhibit cellular respiration and reduce ATP production by binding to functional amino acid groups around the enzyme’s active center, leading to severe ATP depletion and cell membrane potential disorder [13]. It was found that fluoride exposure increased superoxide ion (O^2−^) production, which could modulate the effects of fluoride to a certain extent [14]. In addition, fluoride exposure also increases nitric oxide (NO) in the organism, accumulating misfolded proteins in the endoplasmic reticulum (ER), leading to ER stress and ROS production [15,16]. ROS production leads to mitochondrial damage and DNA damage. In order to counteract this damage, the organism induces the JNK signaling pathway through SIRT1/autophagy to protect cells/ameloblasts from oxidative damage caused by fluoride exposure [17], and excessive autophagy under fluoride exposure will lead to apoptosis [18]. Fluoride exposure induces autophagy but not autophagosome formation and degradation [19]. Fluoride exposure induces mitophagy in mouse testicular interstitial cells through the PINK/Parkin pathway [20]. Studies have demonstrated that fluoride exposure can also induce alterations in cell-cycle progression. Excessive accumulation of fluoride in vivo reduces the number of cells in the G2/M phase of the rat cell cycle [21], causes cycle arrest in the G0/G1 phase of chicken kidney cells [22], and interferes with the progression of G1/S and G2/M phases of rat kidney. However, the existing studies on the mechanism of fluoride exposure in organisms are still very unclear and ambiguous, and there are still many directions worthy of our further exploration.

As one of the most vital organs of silkworm, the silk glands plays a pivotal role in a series of complex biological processes, including the synthesis and secretion of silk proteins, and they represent one of the core focuses related to the silkworm’s economic value [23]. Meanwhile, owing to its high sensitivity to chemical agents, silkworm serves as an ideal model organism for toxicological research. It features low rearing costs, circumvents many ethical concerns associated with mammalian experiments, and has a well-characterized genetic background [24]. Also, there is a basis for research on fluorosis in silkworms. Studies have shown that fluoride ions accumulate first in the midgut after the silkworm consumes fluoride-contaminated mulberry leaves and then diffuse from the intestinal wall to the blood and then to other tissues and organs via the blood [25]. Fluoride exposure adversely affects the growth and development [26], blood [27], midgut [25,28], fat body [28], and gonads [29] of a silkworm. Fluoride uptake by silkworms is keen. When the fluoride content in mulberry leaves is 10 mg/kg, the fluoride content in silkworms can reach 20 mg/kg, which is approximately twice the fluoride content of mulberry leaves that can be accumulated [30]. It has been reported that mulberry leaves soaked in sodium fluoride (NaF) solutions (25, 50, 100, and 200 mg/L) for 15 min and air-dried naturally contained fluoride concentrations of 12.82, 33.95, 67.75, and 208.42 mg/kg, respectively [26].

To better investigate the potential mechanisms of fluoride effects on organisms, we focused on the silk glands of the silkworm, which grow rapidly during the late larval growth period and can grow about 100-fold in just seven days. Therefore, we performed RNA-seq and LC-MS/MS to compare the silk glands of silkworms under fluoride exposure and normal rearing, respectively. DEGs and DEPs were analyzed to find the genes related to the mechanism of fluoride exposure action. The results showed that DEGs and DEPs were significantly enriched in the Ribosome biogenesis in eukaryotes, Protein export, and Protein processing in endoplasmic reticulum pathways. There are 12 overlapping DEGs/DEPs, among which *BmHEATR1*, *BmSPCS2*, *BmSPCS3*, and *BmHsp20.8* are the focus of our attention in the above three pathways.

## 2. Materials and Methods

### 2.1. Silkworm Strains and Fluoride Exposure

For this study, 734, a recognized fluorine-sensitive silkworm strain, was maintained at the Chongqing Sericulture Science and Technology Research Institute, Chongqing, China. One-to-four-instar silkworm larvae were fed mulberry (*Morus*) leaves three times a day in a 12 h light/dark period at 25 ± 1 °C [31]. Fifth-instar silkworm larvae were divided into four groups: one control group and three experimental groups. Each group was set up with three independent replicate zones, with 50 silkworm larvae reared per zone. Silkworm larvae of the experimental groups were fed mulberry leaves soaked in 100 mg/L, 200 mg/L, and 400 mg/L NaF (Kelong, Chengdu, Sichuan, China) solution at the fifth-instar stage (The mulberry leaves were soaked in NaF solution for 15 min followed by air-drying). The control group was given regular mulberry leaves. At sampling, the three replicates of both the control and experimental groups were derived separately from the three independent replicate zones. For transcriptomic and proteomic analyses, one silkworm larva was collected from each replicate zone of the control and 200 mg/L experimental groups, and its silk gland was used as the material for sequencing. No deliberate selection was performed at sampling; the healthiest and the least healthy larvae were excluded, and sampling was performed randomly among the remaining individuals of intermediate condition.

### 2.2. Tissue Sample Collection and Hematoxylin-Eosin Stain (H&E-Stain)

After exposure for six days, silkworm larvae were sacrificed, and silk gland samples from larvae were collected. The samples were stored in −80 °C and 4% paraformaldehyde, respectively. The morphology of some samples was observed immediately. After 24 h, paraformaldehyde was removed, and the posterior silk glands were dehydrated by the gradient (from 75% to 95%) with alcohol (Kelong, Chengdu, Sichuan, China), then cleared with xylene (Chuandong, Chongqing, China) and embedded in paraffin (the melting point is 54–56 degrees, Huakang, Nantong, Jiangsu, China). After paraffin embedding, serial sections were made with a thickness of 5 μm, and then stained with H&E (Sangon, Shanghai, China). Finally, the sections were mounted and observed by electron microscopy (Leica, Wetzlar, Germany) [32].

### 2.3. Transcriptome Sequencing and Analysis

#### 2.3.1. RNA Extraction and Qualification

The total RNA of the experimental and control group was extracted using Trizol Reagent (Invitrogen, Carlsbad, CA, USA) or RNeasy Mini Kit (Qiagen, Hilden, Germany). RNA degradation and contamination were monitored on 1% agarose gels. RNA purity was checked using the NanoPhotometer^®^ spectrophotometer (IMPLEN, Munich, Germany). (OD_260/280_ = 2.0~2.1).

#### 2.3.2. Library Preparation for Transcriptome Sequencing

A total amount of 1 µg RNA per sample was used as input material for the RNA sample preparations. Sequencing libraries were generated using NEBNext^®^ Ultra™ RNA Library Prep Kit for Illumina^®^ (NEB, Ipswich, MA, USA) following the manufacturer’s recommendations, and index codes were added to attribute sequences to each sample. The library was constructed by mRNA purification, fragmentation, first-strand cDNA synthesis, second-strand cDNA synthesis, adaptor ligation, library purification, and library amplification. At last, PCR products were purified (AMPure XP system), and library quality was assessed on the Agilent Bioanalyzer 2100 system and quantified by Qubit 2.0 Fluorometer (Invitrogen, Carlsbad, CA, USA).

#### 2.3.3. Clustering and Sequencing (Novogene Experimental Department)

According to the manufacturer’s instructions, the index-coded samples were clustered on a cBot Cluster Generation System using TruSeq PE Cluster Kit v3-cBot-HS (Illumina, San Diego, CA, USA). After cluster generation, the library preparations were sequenced on an Illumina Hiseq platform, and paired-end reads were generated (Origin-gene Bio-pharm, Wuxi, Jiangsu, China).

#### 2.3.4. Data Analysis

The clean reads were mapped to the silkworm genome database (http://silkbase.ab.a.u-tokyo.ac.jp/cgi-bin/index.cgi (accessed on 18 April 2023)). DEGseq2 [33] was used to analyze differentially expressed genes (DEGs) in the silk gland of the experimental group and the control group, with the standard of |log2(Fold Change)| (|log2FC|) > 1 and adjusted *p* values (*p*-adj) < 0.05. Also, Gene Ontology (GO) and Kyoto Encyclopedia of Genes and Genomes (KEGG) analyses were carried out, with the standard of *p*-adj < 0.05.

### 2.4. Proteome Sequencing and Analysis

#### 2.4.1. Protein Extraction and Digestion

SDT (4% SDS, 100 mM Tris-HCl, 1 mM DTT, pH7.6) buffer was used to lysate silk gland samples and extract protein in the experimental group and the control group. The protein content was quantified by a BCA protein assay kit (Bio-Rad, Hercules, CA, USA). Proteins were digested with trypsin according to the filter-aided sample preparation (FASP) procedure described by Matthias Mann. Digested peptides from each sample were desalted on C18 Cartridges (Empore™SPE Cartridges C18 (standard density), bed I.D. 7 mm, volume 3 mL, Sigma, St. Louis, MO, USA), concentrated by vacuum centrifugation, and reconstituted in 40 μL 0.1% (*v*/*v*) formic acid. For each sample, 20 µg protein was mixed with 5X loading buffer and boiled for 5 min. Proteins were separated on 12.5% SDS-PAGE gel (constant current 14 mA, 90 min). Coomassie blue R-250 staining showed protein bands. Each sample was labeled with a 100 μg peptide mixture using a TMT reagent according to the manufacturer’s instructions (Thermo Scientific, Shanghai, China). The labeled peptides were fractionated with a High pH Reverse-Phase Peptide Fractionation Kit (Thermo Scientific, Shanghai, China).

#### 2.4.2. LC-MS/MS Analysis

LC-MS /MS analysis was performed on a Q Exactive mass spectrometer (Thermo Scientific) coupled to Easy nLC (Proxeon Biosystems, now Thermo Fisher Scientific) for 60/90 min. The peptides were loaded onto a reverse phase trap column (Thermo Scientific Acclaim PepMap100, 100 μm × 2 cm, nanoViper C18) attached to a C18 -reverse-phase assay column (Thermo Scientific Easy Column, 10 cm long, 75 μm inner diameter, 3 μm resin) in buffer A (0.1% formic acid) to buffer the linear gradient separation of B (84% acetonitrile and 0.1% formic acid). MS data were collected using the Top10 data-dependent methods. The instrument was run with peptide recognition mode enabled. MASCOT engine (Matrix Science, London, UK; Version 2.2) was used to search the raw MS data of each sample embedded in Proteome Discoverer 1.4 software for identification and quantitative analysis.

#### 2.4.3. Data Analysis

Cluster 3.0 (http://bonsai.hgc.jp/~mdehoon/software/cluster/software.htm (accessed on 17 October 2022)) and Java Treeview 1.1.6r4 software (http://jtreeview.sourceforge.net) were used to perform hierarchical clustering analysis. Protein sequences were searched using the InterProScan software 5.56-89.0 to identify protein domain signatures from the InterPro member database Pfam. GO terms were mapped, and sequences were annotated using the software program Blast2GO Basic 6.0. The studied proteins were blasted against the online KEGG database (http://geneontology.org/ (accessed on 20 October 2022)) to retrieve their KEGG orthology identifications and were subsequently mapped to pathways in KEGG, with the standard of Fold Change (FC) > 1.2, *p* value < 0.05. For the initial proteomic analysis, we applied a stringent threshold of FC > 2 *p*-adj < 0.05, which yielded only a limited number of differentially abundant proteins. Therefore, a less-stringent cutoff was subsequently used to explore potential underlying biological pathways.

### 2.5. Quantitative Real-Time Polymerase Chain Reaction (RT-qPCR) Analysis

RNAiso Plus (TaKaRa, Dalian, Liaoning, China), chloroform, isopropyl alcohol, 75% ethanol, and DEPC (Sangon, Shanghai, China) were used to extract the total RNA of the experimental group and the control group. Reverse transcription to cDNA was performed by M-MLV reverse transcriptase (TaKaRa, China). cDNA for qPCR analysis of candidate genes with NovoStart^®^SYBR qPCR SuperMix Plus (Novoprotein, Suzhou, Jiangsu, China). The relative expression rates of housekeeping genes *Actin3* were compared, and the relative expression rates of target genes were calculated by 2^−ΔΔ^Ct [34]. Data were analyzed using *t*-tests. Primers were designed with Primer 6.0, and primer sequences were shown in Table S1.

## 3. Results

### 3.1. Effect of NaF on the Development and Morphology of the Silk Gland of the Silkworm

**Figure 1:**
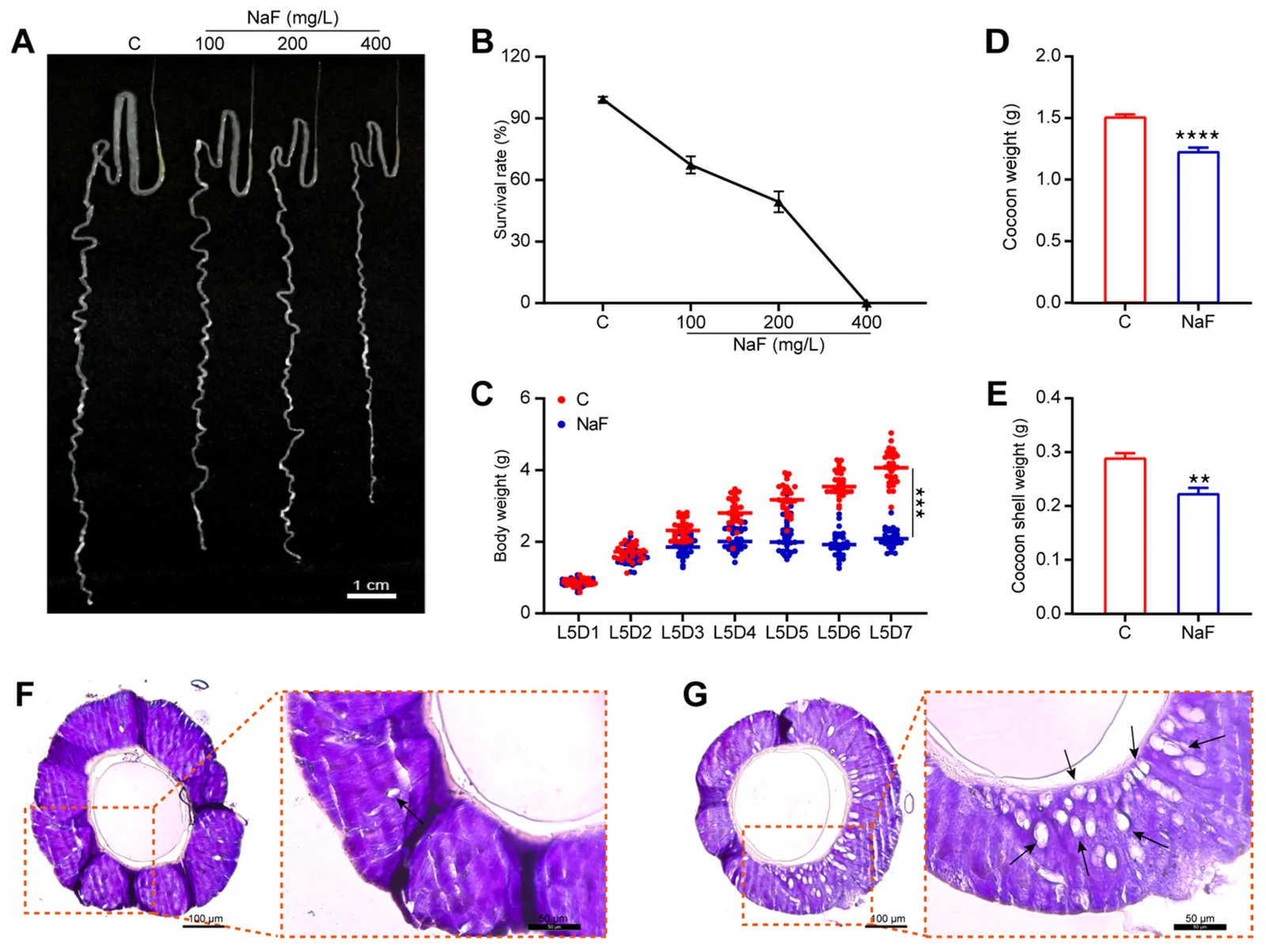
Effects of NaF exposure on silk gland, cocoon, survival rate, and fifth-instar larvae’s weight gain. (**A**) The morphology of silk gland at different concentrations of exposure. C: the control group; 100: the experimental group of 100 mg/L; 200: the experimental group of 200 mg/L; 400: the experimental group of 400 mg/L. The silk glands from larvae treated with 200 mg/L and 400 mg/L NaF were markedly smaller than those of the control group. (**B**) The survival rates of the control group and the experimental group of 100 mg/L, 200 mg/L, and 400 mg/L. *n* = 50. L5D1-L5D7: silkworm larvae from the first day of the fifth instar to the seventh day of the fifth instar. n = 30. At 400 mg/L NaF, all larvae failed to cocoon owing to metamorphosis-associated death. Only half of the larvae were able to form cocoons under the 200 mg/L NaF treatment. (**C**) The weight trends in the control group and the experimental group. C: the control group, indicated by red solid circles; NaF: the experimental group of 200 mg/L, indicated by blue solid circles. L5D1, L5D2, L5D3, L5D4, L5D5, L5D6, and L5D7: the fifth larva stage’s 1st, 2nd, 3rd, 4th, 5th, 6th, and 7th day. *n* = 30. The body weight of larvae in the control group increased normally, whereas the body weight of experimental group larvae showed no obvious weight gain after L5D4. ***: *p*< 0.001. (**D**,**E**) The cocoon and cocoon shell weights of the control and experimental groups. *n* = 20 (There are 10 female pupae and 10 male pupae). C: the control group; NaF: the experimental group of 200 mg/L. Both cocoon weight and cocoon shell weight were highly significantly lower in the experimental group compared with the control group. **: *p* < 0.01; ****: *p* < 0.0001. (**F**,**G**) The morphology (H&E staining) of silk gland at the control (**F**) and experimental (**G**) group. The right side is a magnification of the red box on the left. The arrow points to the intercellular vacuoles. *n* = 3. Vacuoles were markedly more abundant in the experimental group than in the control group.

To investigate the effects of fluoride exposure dose and duration on the growth and development of fifth-instar silkworm larvae, we performed a series of feeding experiments. NaF exposure concentration affected silk gland development, and increasing NaF concentrations substantially retarded silk gland growth. Silk glands isolated from larvae exposed to 200 mg/L and 400 mg/L NaF were significantly smaller compared with control silk glands (Figure 1A). The survival rate of fifth-instar larvae declined significantly with rising of NaF concentration (Figure 1B). When treated with 400 mg/L NaF, all larvae failed to cocoon and died due to blocked metamorphosis. Only half of the larvae successfully produced cocoons at the 200 mg/L NaF exposure level. Therefore, for the sake of continuity of the subsequent experiments and validity of the experimental effects, the silkworm reared with mulberry leaves soaked in 200 mg/L NaF was selected as the experimental group in this study. Figure 1C shows the body weight growth rate of the fifth-instar larvae of the silkworm. With the increase in exposure time, the control group of silkworm larvae grew as usual. The experimental group of silkworm larvae had no significant weight growth after the fourth day of the fifth instar. The cocoon and cocoon shell weights of the experimental group were significantly smaller than those of the control group (Figure 1D,E). Paraffin sections of the posterior silk glands of the silkworm showed that the cells of the posterior silk glands in the experimental group were loosely arranged and had more intercellular vacuoles than those in the control group (Figure 1F,G).

### 3.2. Transcriptome Changes

#### 3.2.1. Differentially Expressed Genes (DEGs) in the Experimental Groups

**Figure 2:**
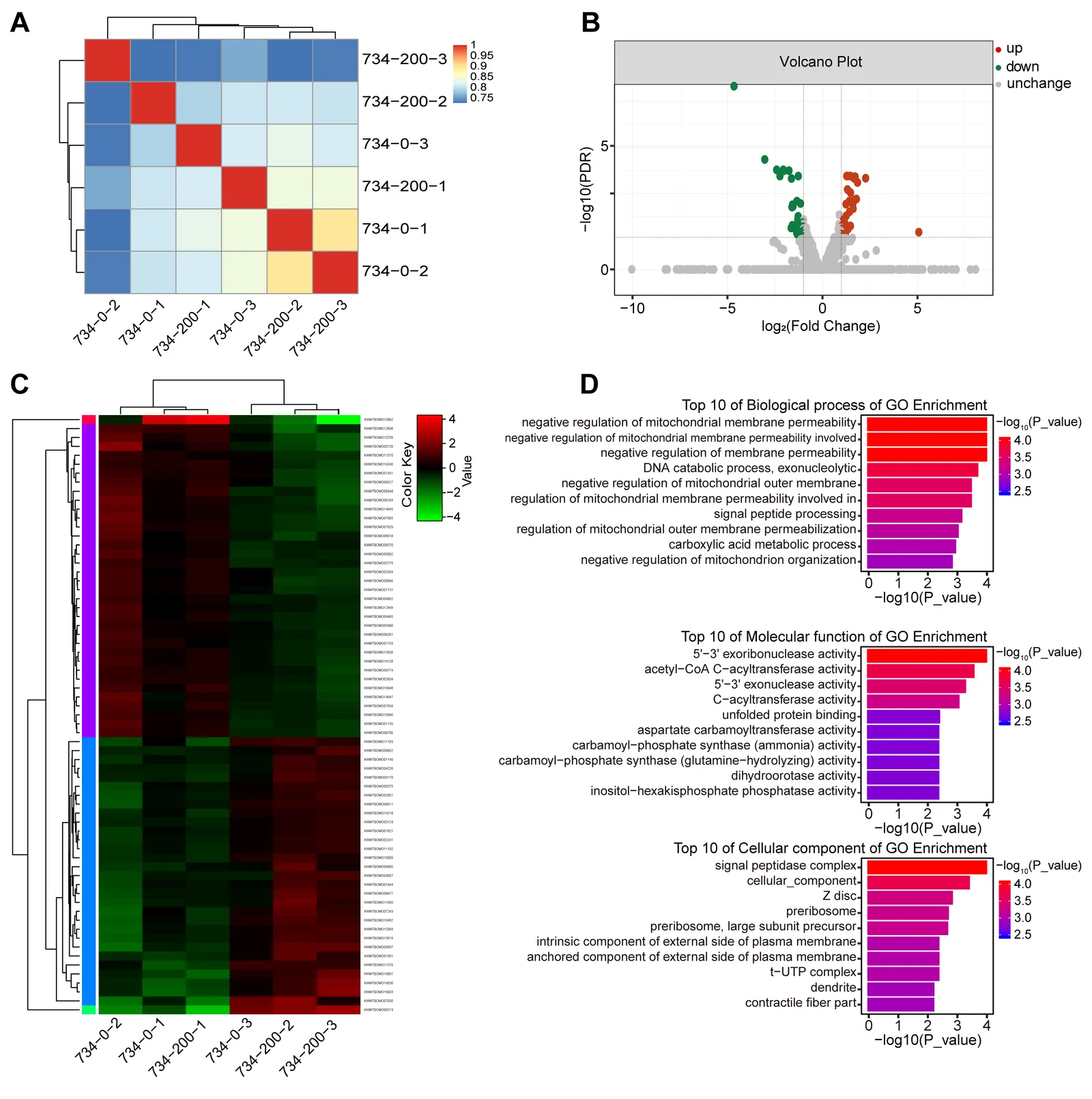
Transcriptome data and GO enrichment analysis. (**A**) Pearson correlation (R^2^) analysis of samples. The number represented by the color was the correlation coefficient between each two samples. (**B**) Volcano plots of the DEGs. Red dots indicate up-regulated genes, green dots indicate down-regulated genes, and gray dots indicate the genes that have not changed significantly. (**C**) The heatmap of the control and experimental groups. Green indicates low expression, black indicates moderate expression, and red indicates high expression. (**D**) Significant enrichment of GO in DEGs. *n* = 3.

To explore the potential mechanisms of fluoride effects on the silk glands of the silkworm, RNA-Seq was used to analyze the transcription level of silk glands. The quality control parameters of the RNA-Seq data are shown in Table S2. Three transcriptome libraries were constructed for each experimental and control group. Each transcriptome library generated between 42 and 57 million raw reads. The analysis’s quality score showed that the data quality was robust. Correlation analysis was performed on the DEGs to analyze the expression patterns between and within groups. The heat map showed that the correlations between and within groups were more significant than 0.75, indicating that there were similar expression patterns between and within groups, with good reproducibility (Figure 2A). Pearson correlation coefficient (R^2^) analysis of the two groups revealed moderate dispersion among biological replicates. Quality-control metrics of all samples met the criteria for downstream analysis. All samples were sequenced in a single batch, which excludes the effects of technical errors and batch effects. Since each biological replicate in our experiment was derived from an individual silkworm larva, we attribute this dispersion phenomenon to the inherent individual variation among silkworm larvae. The two-way clustering heat map shows that both experimental and control groups have good biological replication and also visualizes the expression changes of differential genes (Figure 2C). In order to demonstrate the significant difference in genes between the comparison groups, this study set |log2 FC| > 1 and *p*-adj < 0.05 as the threshold to screen DEGs. In total, 67 DEGs were found through screening, of which 36 were up-regulated and 31 were down-regulated (Figure 2B).

#### 3.2.2. Gene Ontology (GO) and Kyoto Encyclopedia Genes and Genomes (KEGG) Analysis

To understand the functions of DEGs in the silk gland of silkworms after fluoride exposure, we performed functional annotation analysis on DEGs. GO is a standardized functional classification system, which is divided into three main categories: Biological process (BP), Cellular component (CC), and Molecular function (MF). The top 10 items of each category were selected and ranked by *p* value. The top three functions with significant enrichment of BP were negative regulation of mitochondrial membrane permeability, negative regulation of mitochondrial membrane permeability involved in apoptotic process, and negative regulation of membrane permeability, all of which belong to the regulation of membrane permeability. The top three functions with significant enrichment of CC were signal peptidase complex, cellular component, and Z disc. The top three functions of MF significant enrichment were 5′-3′ exoribonuclease activity, acetyl-CoA C-acyltransferase activity, and 5′-3′ exonuclease activity, which are catalytic activities, except acetyl-CoA C-acyltransferase activity, which is a transferase activity; the other two categories are hydrolase activities. Four GO terms are associated with cell membranes, and we hypothesized that fluoride disrupts cell membrane permeability (Figure 2D).

**Figure 3:**
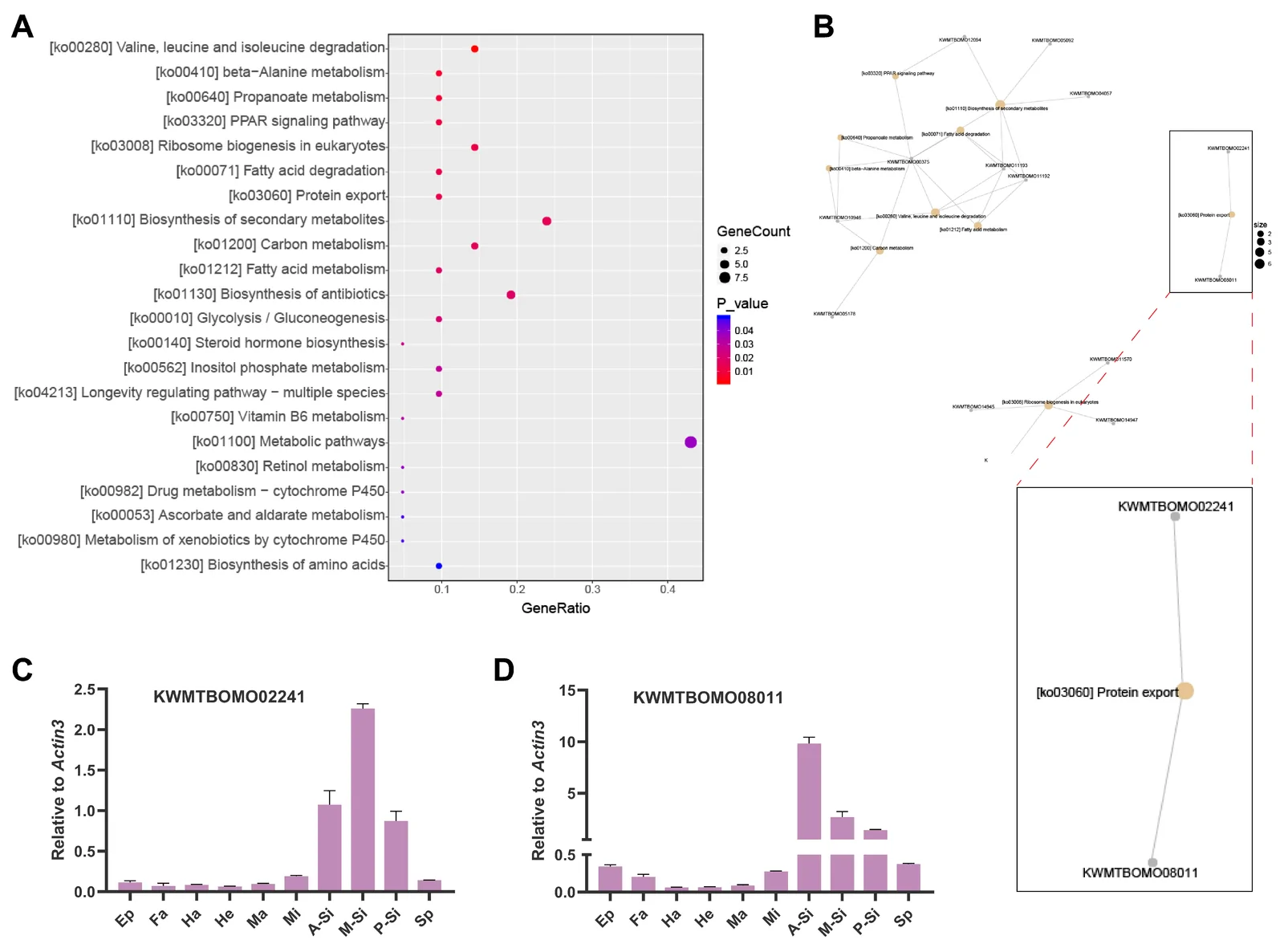
KEGG enrichment analysis. (**A**) Significant enrichment of KEGG in DEGs. *p*-adj < 0.05. *n* = 3 (**B**) Cnetplot of KEGG. The black rectangle box is a partial enlarged figure. Golden nodes represent KEGG pathways, while grey nodes correspond to differentially expressed genes; node size is proportional to the number of enriched genes in each pathway. Pathway annotation data were obtained from the KEGG database (https://www.kegg.jp/ (accessed on 8 May 2023)). (**C**,**D**) Tissue distribution of KWMTBOMO02241 and KWMTBOMO08011. Ep: epidermis, Fa: fat body, Ha: hemocyte, He: head, Ma: Malpighian tube, Mi: midgut, A-Si: anterior division of silk gland, M-Si: middle silk gland, P-Si: posterior division of silk gland, and Sp: spiracle. *n* = 3. These two genes exhibited the highest expression levels in the silk glands.

We mapped the DEGs to the KEGG database to further explore the biological pathways associated with the silk glands of silkworms after fluoride exposure. The KEGG database contains information on multiple pathways in Metabolism, Genetic Information Processing, Environmental Information Processing, Cellular Processes, Organismal Systems, Human Diseases, and Brite Hierarchies. Figure 3A shows the top 22 significant enrichment pathways, of which the PPAR signaling pathway and Longevity regulating pathway—multiple species belong to Organismal Systems, Ribosome biogenesis in eukaryotes, and Protein export belongs to Genetic Information Processing. In contrast, the other pathways all belong to Metabolism. Ribosome biogenesis in eukaryotes is a crucial prerequisite for translating RNA into proteins. It is one of the most multifaceted and energy-intensive processes in biology, involving many assemblies and maturation factors coordinated by multiple cellular inputs [35]. Impairment of this pathway leads to stalled ribosome growth, thus leading to cell termination. Protein export transports newly synthesized proteins to and across the cell membrane, and impairment of this pathway affects protein transport [36]. Cnetplot (Gene-Concept network) shows two significant DEGs of the Protein export pathway were significantly enriched, KWMTBOMO02241 (*BmSPCS2*) and KWMTBOMO08011 (*BmSPCS3*) (Figure 3B). Detection of the tissue expression pattern of these two genes revealed the highest expression in the silk gland (Figure 3C,D).

#### 3.2.3. Gene Set Enrichment Analysis (GSEA) and Verification of Differentially Expressed Genes

**Figure 4:**
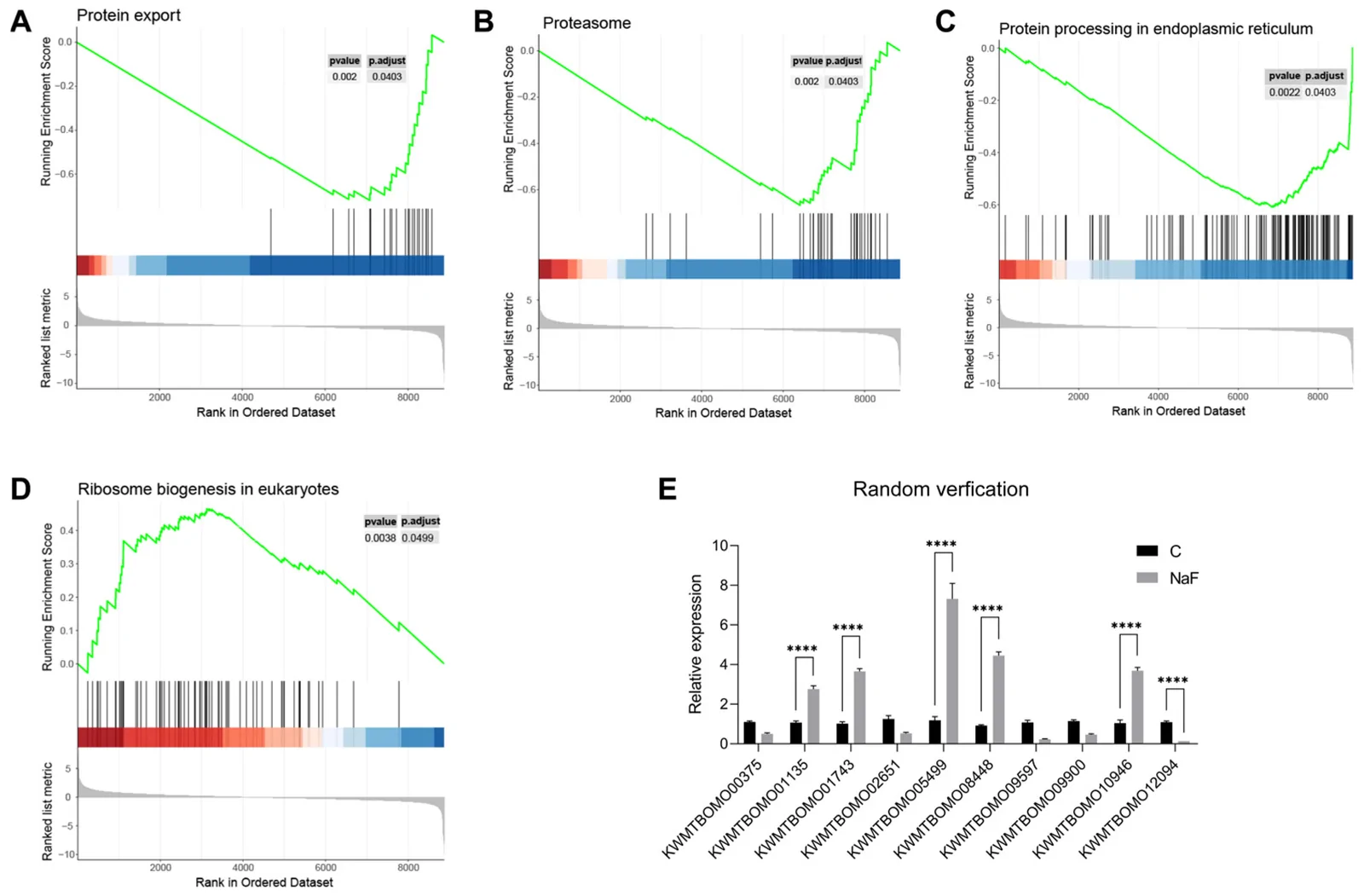
GSEA analysis and verification of differentially expressed genes. (**A**–**D**) GSEA analysis. *p*-adj < 0.05. *n* = 3. Green line: enrichment score (ES); vertical black lines: hit genes in the gene set; red-blue gradient: up/downregulated genes, darker colors = more significant changes. Protein export (**A**), Proteasome (**B**), Protein processing in endoplasmic reticulum (**C**), and Ribosome biogenesis in eukaryotes (**D**). (**E**) Validation of ten random genes by RT-qPCR. C: the control group. NaF: the experimental group. NaF: the experimental group of 200 mg/L. ****: *p* < 0.0001. *n* = 3.

In order to find sets of genes that share standard biological function, chromosomal location, or regulation, we performed a GSEAanalysis on DEGs. GSEA is often used to interpret gene expression data [37]. GSEA enrichment analysis does not rely on a fixed cutoff threshold. It considers the expression trends of all genes, which enables the detection of pathways with coordinated overall changes even when individual genes do not show significant differential expression. The top 15 significantly enriched pathways are shown in Table S3, all *p*-adj < 0.05. Among the enriched GSEA pathways, protein export (Figure 4A), proteasome (Figure 4B), protein processing in endoplasmic reticulum (Figure 4C), and ribosome biogenesis in eukaryotes (Figure 4D) all belong to Genetic Information Processing, which performs protein transport, renewal, processing, and translation. These four pathways were our primary targets for further investigation. For the protein export gene set, the setSize was 22, the enrichment score (ES) was −0.72, and the normalized enrichment score (NES) was −2.06, indicating that a greater number of genes showed down-regulated enrichment. For the proteasome gene set, the setSize was 36, the ES was −0.67, and the NES was −2.12, indicating that a greater number of genes showed down-regulated enrichment. For the protein processing in endoplasmic reticulum gene set, the setSize was 135, the ES was −0.61, and the NES was −2.46, indicating that a greater number of genes showed down-regulated enrichment. For the ribosome biogenesis in eukaryotes gene set, the setSize was 70, the ES was 0.47, and the NES was 1.67, indicating that a greater number of genes showed up-regulated enrichment.

Ten DEGs were randomly selected for validation (Figure 4E), where KWMTBOMO00375, KWMTBOMO02651, KWMTBOMO09597, and KWMTBOMO09900 down-regulated expression, and KWMTBOMO12094 highly significantly down-regulated expression. KWMTBOMO01135, KWMTBOMO0174, KWMTBOMO05499, KWMTBOMO08448, and KWMTBOMO10946 highly significantly up-regulated expression, and the trend was consistent with the transcriptome data. Correlation analysis of the expression levels of the validated random genes with those of the transcriptome genes showed an R^2^ of 0.916 (Figure S1), indicating a strong correlation.

### 3.3. Proteome Changes

#### 3.3.1. Differentially Expressed Proteins (DEPs) in the Experimental Groups

**Figure 5:**
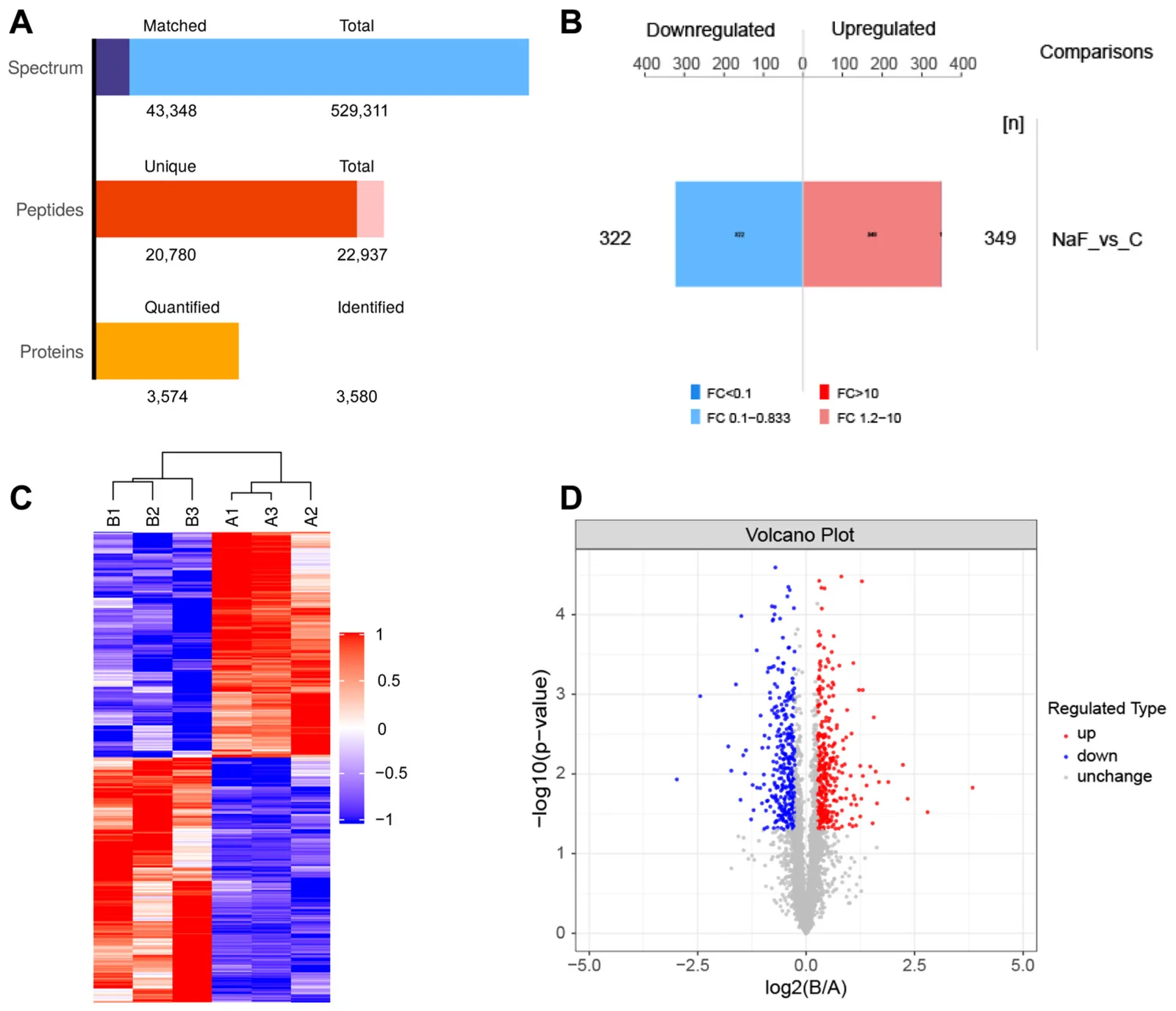
Proteome data. (**A**) Statistical bar chart of identification and quantitative results. Light blue: Total spectrum; Dark blue: Matched spectrum; Light red: Total Peptides; Dark red: Unique Peptides; Light yellow: Identified proteins; Dark yellow: Quantified proteins. (**B**) Histogram of protein quantitative difference results. (**C**) The result of cluster analysis of DEPs. Blue indicates low expression, white indicates no protein quantification information was available, and red indicates high expression. Red: Significantly upregulated proteins; Blue: Significantly downregulated proteins; Blue: Significantly downregulated proteins. (**D**) Volcano plots of the DEPs. Red dots indicate up-regulated proteins, blue dots indicate down-regulated proteins, and gray dots indicate the proteins that have not changed significantly. *n* = 3. FC > 1.2 (up-regulation > 1.2-fold or down-regulation < 0.83-fold, *p* value < 0.05.

In this study, TMT quantitative proteome technique (TMT) was used to analyze the variation in protein expression of silk glands of silkworms fed with regular mulberry leaves and mulberry leaves soaked with 200 mg/L NaF solution. The quality control parameters of the LC-MS/MS-based proteomics plots are shown in Figure S2. The total number of the spectra obtained, the number of peptides identified, and the number of proteins identified in this project are shown in Figure 5A. The number of quantifiable proteins identified was 3574. In order to analyze the proteins with differences between different groups, the experimental data were further screened for differences. With Fold change (FC) > 1.2 (up-regulation > 1.2-fold or down-regulation < 0.83-fold) and *p* value < 0.05 as the criteria, 349 differentially up-regulated proteins and 322 differentially down-regulated proteins were obtained between the comparison groups, totaling 671 DEPs (Figure 5B). Among them, the most significant up-regulated protein, sericin 2 isoform 1 precursor, was up-regulated by 14-fold. The most significant down-regulated protein, XP_004926112.1, was an unknown protein, down-regulated by 0.12-fold. In order to analyze the expression patterns between and within groups, to test the rationality of grouping in this research, and to indicate whether the differential protein expression changes could represent significant effects of biological treatments on the samples, the proteins in the experimental and control groups were grouped using a hierarchical clustering algorithm and presented as a heat map (Figure 5C) with FC > 1.2 and *p* value < 0.05 as the criteria. The obtained significantly differentially expressed proteins can effectively separate the comparison groups, indicating that the differentially expressed protein screening can represent the effect of biological treatments on the samples. To demonstrate the significant differences in proteins between the comparison groups, we drew the volcano map of the proteins in the comparison groups with two factors, FC and *p* value, where significantly down-regulated DEPs are labeled in blue (FC < 0.83, *p* value < 0.05) and significantly up-regulated DEPs are labeled in red (FC > 1.2, *p* value < 0.05) (Figure 5D).

#### 3.3.2. GO and KEGG Analysis

**Figure 6:**
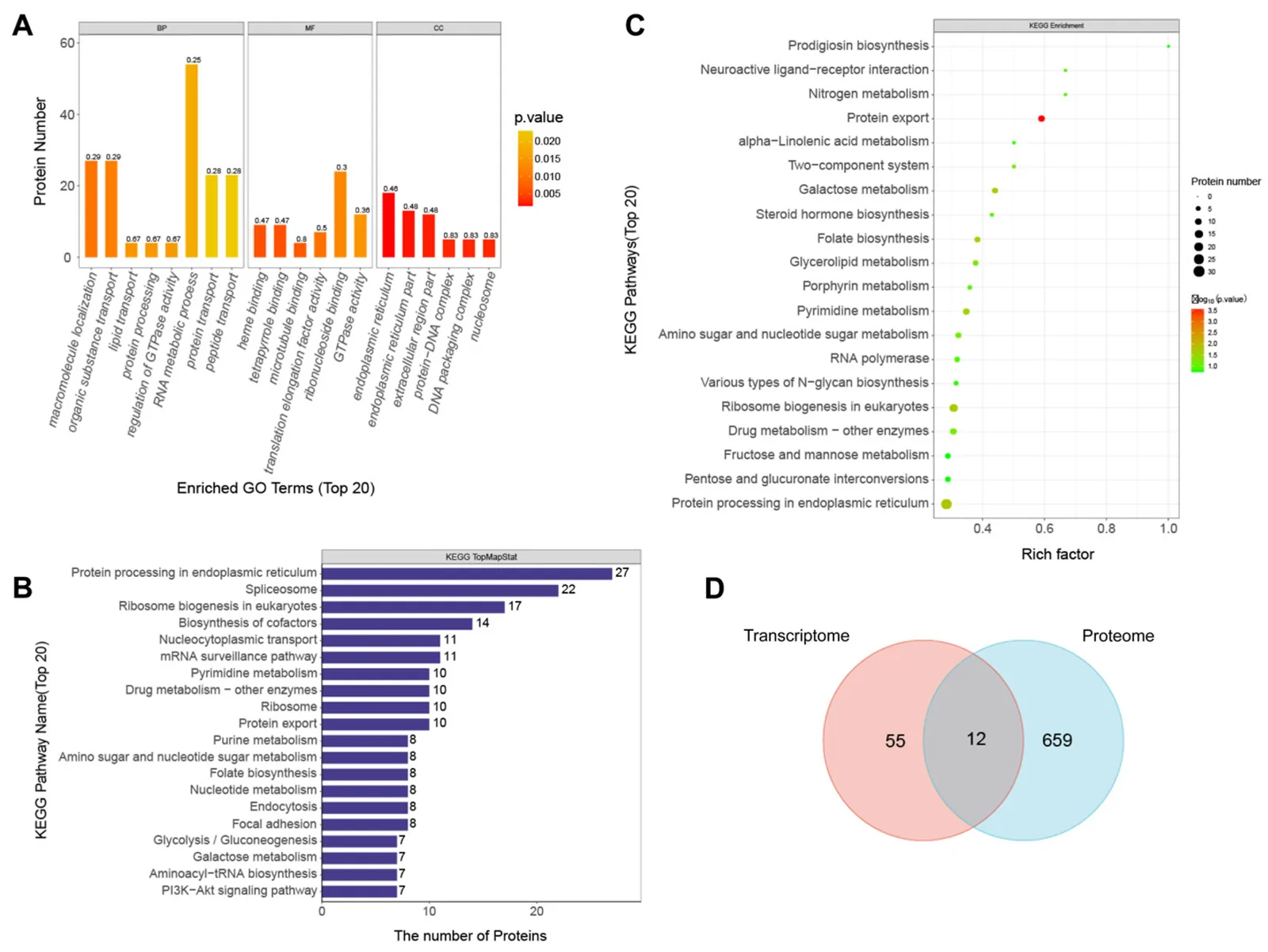
GO and KEGG enrichment analysis. (**A**) Significant enrichment of GO in DEPs. (**B**) Statistical diagram of KEGG pathway annotation for DEPs. (**C**) KEGG pathway enrichment bubble map of DEPs. (**D**) Venn diagram of transcriptome and proteome. *n* = 3. FC > 1.2. *p* value < 0.05.

To understand the function, localization, and biological pathways involved in DEPs in organisms, to reveal the overall functional enrichment characteristics of all DEPs and to find the significantly enriched GO terms of most interest to the study by evaluating the significance level of protein enrichment for a particular GO functional term, we performed a functional enrichment analysis of DEPs. The Fisher’s Exact Test was used to derive the significance of DEPs, thus finding the functional categories in which all DEPs were enriched (*p* value < 0.05) (Figure 6A). In BP, macromolecule localization and organic substance transport were the most significant differences, and RNA metabolic process was enriched with the most DEPs. In MF, heme binding and tetrapyrrole binding were the most significant differences, and ribonucleoside binding enriched the most DEPs. In CC, endoplasmic reticulum enriched the most DEPs with the most significant differences.

In order to analyze the biological process, disease mechanism, and drug action mechanism more systematically and comprehensively, a series of proteins are often needed to elaborate the changing pattern from the perspective of coordinated action. In this study, we performed KEGG pathway analysis and annotation of the DEPs. The top pathways with the highest number of DEPs were Protein processing in endoplasmic reticulum, Spliceosome, Ribosome biogenesis in eukaryotes, Biosynthesis of cofactors, and Nucleocytoplasmic transport (Figure 6B). In order to reveal the overall pathway enrichment characteristics of all DEPs and to find the most significant enriched KEGG pathway to focus on by evaluating the significance level of protein enrichment of a particular KEGG pathway, this research used Fisher’s Exact Test on DEPs for KEGG pathway enrichment analysis (*p* value < 0.05). The results showed that Protein export, Protein processing in endoplasmic reticulum, Galactose metabolism, Ribosome biogenesis in eukaryotes, Folate biosynthesis, and other significant changes were observed in critical pathways (Figure 6C).

### 3.4. Combined Transcriptome and Proteome Analysis

The transcriptome can yield a good deal of differential genes and numerous regulatory networks. However, it is challenging to identify key pathways and characterize the structures that control them. Furthermore, genes generally cannot affect metabolites directly, nevertheless this occurs through translation to proteins and then to metabolites, which involves the proteome. In this research, the transcriptome and proteome were jointly analyzed, and genes and proteins were mutually verified to profoundly investigate the biological processes and biological network regulation that affect the silk glands of silkworms under fluoride exposure. The analysis revealed that 67 DEGs were identified in the transcriptome and 671 DEPs in the proteome. A Venn diagram showed that only 12 differentially expressed genes/proteins were overlapping between the two sets of omics in the transcriptional and protein levels (Figure 6D). Of these 12 shared differentially expressed genes/proteins, the expression trends were all consistent, with three expressions up-regulated and nine expressions down-regulated. Among these 12 DEGs/DEPs, KWMTBOMO01743/XP_037866437.1 (BmHEATR1) exists in Ribosome biogenesis in eukaryotes (ko03008). KWMTBOMO02241/NP_001040214.1 (BmSPCS2), KWMTBOMO08011/NP_001091763.1 (BmSPCS3) were present in Protein export (ko03060). KWMTBOMO16003/NP_001091794.1 (BmHsp20.8) were present in Protein processing in endoplasmic reticulum (ko04141) (Table S4).

**Figure 7:**
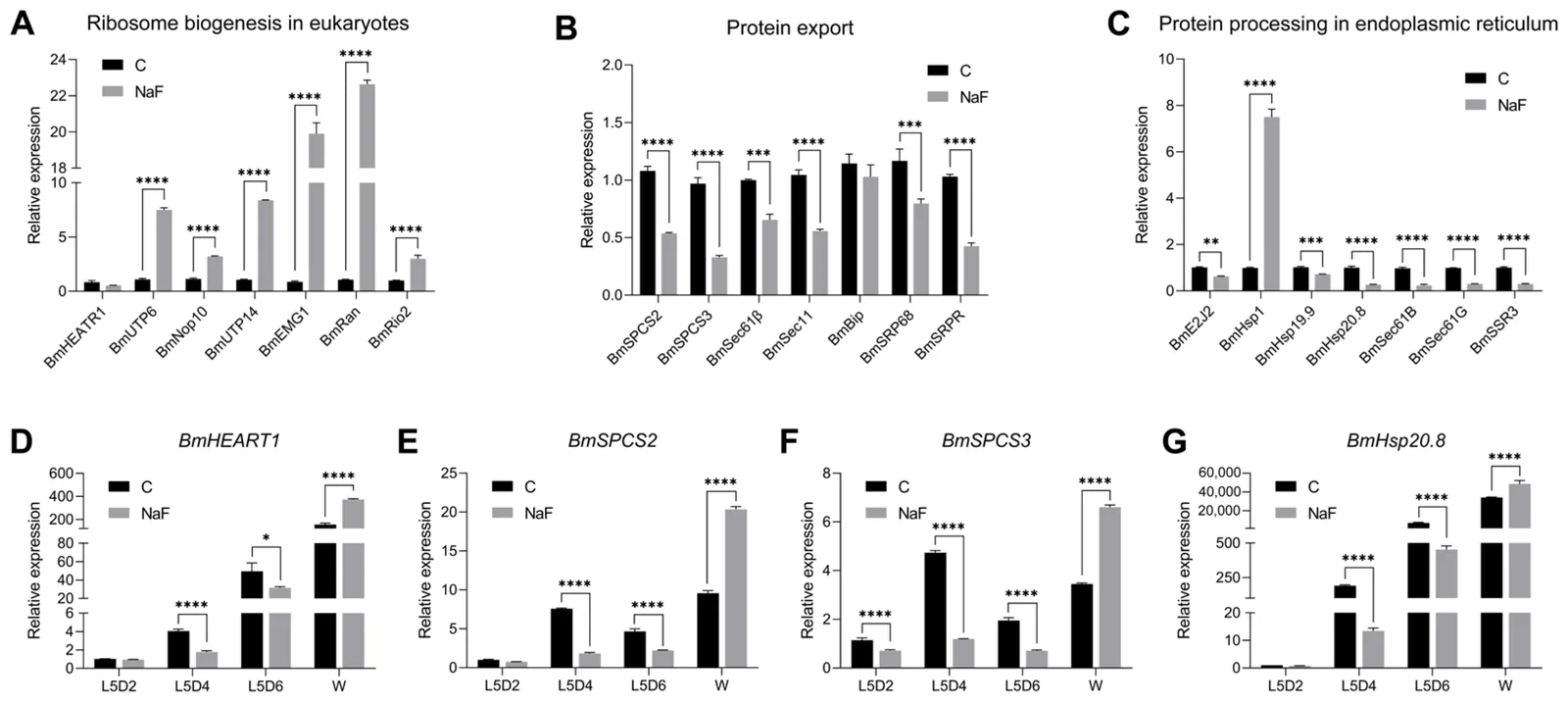
RT-qPCR validated the overlap pathways and genes/proteins of mRNA and protein levels. (**A**) Validation of Ribosome biogenesis in eukaryotes by RT-qPCR. *BmHEATR1* was down-regulated, whereas *BmUTP6*, *BmNop10*, *BmUTP14*, *BmEMG1*, *BmRan*, and *BmRio2* were highly significantly up-regulated. (**B**) Validation of Protein export by RT-qPCR. *BmBip* was down-regulated, and *BmSPCS2*, *BmSPCS3*, *BmSec61β*, *BmSec11*, *BmSRP68*, and *BmSRPR* were highly significantly down-regulated. (**C**) Validation of Protein processing in endoplasmic reticulum by RT-qPCR. *BmHsp1* was highly significantly up-regulated, whereas *BmR2J2*, *BmHsp19.9*, *BmHsp20.8*, *BmSec61B*, *BmSec61G*, and *BmSSR3* highly significantly down-regulated. (**D**–**G**) The expression of *BmHEATR1*, *BmSPCS2*, *BmSPCS3*, and *BmHsp20.8* at the L5D2, L5D4, L5D6, and W by RT-qPCR. All four genes showed significant down-regulation at L5D4 and L5D6, whereas their expression was significantly up-regulated during the W. C: the control group. NaF: the experimental group of 200 mg/L. *n* = 3. RNA was extracted from posterior silk glands. L5D2, L5D4, and L5D6: the fifth larva stage’s 2nd, 4th, and 5th day. W: Wandering period (Thorax transparent silkworm; the larva is about to start spinning and forming cocoons). *: *p* < 0.05; **: *p* < 0.01; ***: *p* < 0.001; ****: *p* < 0.0001.

Then we validated the overlap pathways and genes/proteins of mRNA and protein levels. DEGs in Ribosome biogenesis in eukaryotes were selected for validation (Figure 7A), in which *BmHEATR1* down-regulated expression, while *BmUTP6*, *BmNop10*, *BmUTP14*, *BmEMG1*, *BmRan*, and *BmRio2* highly significantly up-regulated expression. The trends were broadly consistent with the transcriptome data (Table S5). DEGs in Protein export were selected for validation (Figure 7B), in which *BmBip* down-regulated expression, *BmSPCS2*, *BmSPCS3*, *BmSec61β*, *BmSec11*, *BmSRP68*, and *BmSRPR* highly significantly down-regulated expression. The trends were broadly consistent with the transcriptome data (Table S6). DEGs in Protein processing in endoplasmic reticulum were selected for validation (Figure 7C), in which *BmE2J2* significantly down-regulated expression, *BmHsp19.9*, *BmHsp20.8*, *BmSec61B*, *BmSec61G*, and *BmSSR3* highly significantly down-regulated expression. At the same time, *BmHsp1* was highly significantly up-regulated expression. The trends were consistent with the transcriptome data (Table S7).

The control and the experimental groups enabled the detection of the expression levels of critical genes *BmHEATR1*, *BmSPCS2*, *BmSPCS3*, and *BmHsp20.8* in the above three pathways. Compared with the control group, the expression of *BmHEATR1* is as follows (Figure 7D). The expression was down-regulated at L5D2. The expression of L5D4 was highly significantly down-regulated. The expression was significantly down-regulated at L5D6. Strangely, in the W, the expression level was highly significantly up-regulated. The expression of *BmSPCS2* and *BmSPCS3* were slightly different (Figure 7E,F). At L5D2, the expression of *BmSPCS3* was highly significantly down-regulated, and there was no significant change in *BmSPCS2*. Both *BmSPCS2* and *BmSPCS3* were highly significantly down-regulated in L5D4 and L5D6. However, in the W, both genes were highly significantly up-regulated. The expression of *BmHsp20.8* had significant changes except in L5D2 (Figure 7G). The expression of *BmHsp20.8* was highly significantly down-regulated at L5D4 and L5D6. At the W, *BmHSP20.8* was highly significantly up-regulated.

## 4. Discussion

Fluoride, as a cumulative toxicant, can affect the growth and development of organisms and cause physiological and pathological damage when it is widely present in the atmosphere causing pollution. In silkworms, previous transcriptome studies comparing midgut fluoride-tolerant and fluoride-susceptible silkworm varieties have revealed that genes related to ribosome and glutathione metabolism pathways exert substantially stronger transcriptional responses in fluoride-susceptible silkworm individuals [38]. Reported transcriptome studies of the midgut and fat body under fluoride exposure showed that fluoride exposure significantly inhibited oxidative phosphorylation processes, impairing ATP synthesis, and stimulating the MAPK signaling pathway through phosphorylation signaling [28]. However, it is difficult to explain the complex biological processes and network regulation by single histological data alone. Integrating multi-omics data for analysis can remedy the data problems caused by missing data and noise during single histology data analysis. In addition, existing research concerning fluoride stress in silkworms mainly targeted the midgut [28,38], fat body [28] and testis [26,29]; few investigations have been reported on the silk gland, the core tissue determining the economic value of silkworms. Therefore, in the present study, we focused on the differential changes of the transcriptome and proteome in silkworm silk glands to better elucidate the action mechanism of fluoride exposure on silk glands. The target genes/proteins involved in the process of fluoride exposure were identified in the analysis of DEGs/DEPs, and KEGG and GO enrichment analysis identified critical pathways in the process of fluorine exposure.

Firstly, we observed Ribosome biogenesis in eukaryotes. This pathway was significantly enriched not only in transcriptome KEGG, GSEA, but also in proteome KEGG. KWMTBOMO01743/XP_037866437.1 (*BmHEATR1*) was significantly up-regulated expression. Ribosomes are one of the most complex molecular structures in biology, and their biogenesis is one of the most complex biological processes. Ribosomes translate information in mRNA into functional proteins, the final step in the genetic program [39]. Most of the steps of ribosome biogenesis occur in the nucleolus. The mature 80S ribosome consists of a 40S subunit and a 60S subunit. Ribosomal DNA (rDNA) transcription of 47S precursor rRNAs (47S pre-rRNAs) is relevant [40,41]. The 47S pre-rRNAs co-transcribe and assemble with 5S rRNA to form the 90S processome. The 90S processome binds to ribosomal proteins (RPs) to mature into pre-40S and pre-60S ribosomal subunits [42]. Of these, mRNA and tRNA enter the cytoplasm from the nuclear pore, bind to ribosomes in the cytoplasm, are translated into RPs, and reenter the nucleolus to bind to the 90S processome to form precursor ribosomes. The last two subunits enter the cytoplasm from the nuclear pore and bind as mature 80S ribosomes for the translation step [43]. Overactive ribosome biogenesis has been implicated as a hallmark of many cancers and is associated with poor prognosis [35]. Perturbation of ribosome biogenesis, leading to the activation of the p53 tumor suppressor protein, promotes cell cycle arrest, apoptosis, or senescence [44,45]. Studies have shown that protein synthesis is essential for cell growth. Up-regulation of ribosomal proteins mirrors rapid silk gland growth, and intensified ribosome biogenesis is key to efficient silk protein synthesis [46]. *HEATR1* (a member of the *UTP10* family, it is named *BmHEATR1* in *Bombyx mori*) is a member of the t-UTP complex, and t-UTPs are required for efficient transcription of rDNA and are relatively conserved over evolutionary time [47]. Previous studies have shown that *HEATR1* induces specific cytotoxic T lymphocyte (CTL) responses against A2B5+ glioblastoma (GBM) cells and glioma stem-like cells (GSC) [48]. *HEATR1* deficiency promotes pancreatic cancer proliferation and gemcitabine resistance by up-regulated *Nrf2* [49]. Deficiency of *HEATR1* leads to perturbation of ribosome biogenesis, resulting in non-dependent activation of DNA damage through the RPL53/RPL21-MDM5 axis activating the p11-p2 checkpoint response [50]. Therefore, we hypothesize that fluoride accumulation in the silkworm’s silk glands causes up-regulated *BmHEATR1* expression to disrupt ribosome biogenesis, severely affecting protein translation.

Then, we focused on Protein export, a pathway significantly enriched in the transcriptome KEGG, GSEA, and the proteome KEGG. KWMTBOMO02241/NP_001040214.1 (*BmSPCS2*) and KWMTBOMO08011/NP_001091763.1 (*BmSPCS3*) were significantly down-regulated expression. Protein export is a pathway for protein translocation from the cytoplasmic matrix to the lumen of the endoplasmic reticulum (ER). The entire process by which this pathway occurs is known as the signal hypothesis. The nascent polypeptide chains in the free ribosome isolate signal peptides (SPs), which are recognized and bound by signal recognition particles (SRPs). At this point, the protein stops translation to prevent damage to the N-terminal end of the nascent peptide chain and to fold prior to maturation [51]. The signal recognition particle receptor (SRPR) on the ER membrane interacts with the SRP. The ribosome and nascent chain are transferred to a protein conduction channel (SecYEG complex for prokaryotes and Sec61 complex for eukaryotes) bound to the SRPR [52,53]. After ribosome binding to the Sec61 complex, the SRP breaks away from the SPs and enters the cytoplasmic matrix for reuse. At this point, the translation process restarts, and as translation continues, the translocation subchannel opens, and the SPs guide the peptide chain into the lumen of the ER in a semi-loop. At the same time, signal peptidase (SPC) located on the ER membrane excises the SPs and rapidly degrades the SPs. The peptide chain continues to extend into the ER lumen until the completion of the entire peptide chain [54,55]. The SPC functions in this pathway to separate the SPs from their nonfunctional precursors to release mature proteins from the membrane or secretory pathway [56]. The SPC is an essential membrane complex in the ER. The SPC of the human ER has five subunits, including *Sec11A*, *Sec11C*, *SPCS1*, *SPCS2* (It is named *BmSPCS2* in *Bombyx mori*), and *SPCS3* (It is named *BmSPCS3* in *Bombyx mori*) [56]. *Sec11A* is a prognostic marker in cancer patients with resected cancerous tissue [57]. Studies of the signal peptide processing pathway required by flaviviruses have revealed that *SPCS1* preferentially promotes the processing of specific protein cargoes directly or indirectly through its interaction with unknown host proteins [58]. Studies of trophoblast function during Zika virus infection revealed that *SPCS1* is an essential Zika virus host factor in placental trophoblasts [59]. It has been reported that Signal peptide peptidase (*BmSPP*) exerts negative-regulation effects on silkworm resistance against BmNPV infection and participates in the processes of viral proliferation and replication [60]. We hypothesize that the accumulation of excess fluoride in the silkworm’s silk glands down-regulated the expression of *BmSPCS2* and *BmSPCS3* and affected the isolation of SPs, thus leading to the inability of polypeptide chains to enter the ER lumen smoothly and accumulate on the ER membrane, which in turn affects protein folding and assembly.

Finally, we focused on Protein processing in endoplasmic reticulum, a pathway significantly enriched in transcriptome GSEA and proteome KEGG. KWMTBOMO16003/NP_001091794.1 (*BmHsp20.8*) in this pathway was significantly down-regulated in both histological assays. The polypeptide chain enters the ER lumen unfolded and remains an immature protein. Folding takes place with the help of luminal chaperones in the ER. However, various physiological or pathological stresses accumulate misfolded proteins in the ER, which triggers ER stress and activates the unfolded protein response (UPR) [61]. Proteome results showed significantly up-regulated PERK protein expression in UPR, with no significant changes in ATF6 and IRE1. Protein quality control in ER (ERQC), besides accelerating adequate protein folding, impacts UPR also through ER-associated degradation (ERAD) [62]. ERAD requires the movement of the peptide from the ER resident site into the cytoplasm, followed by ubiquitination and further extraction into the cytoplasm via the p97 complex, and finally, degradation by the proteasome to a short peptide [63]. *Hsp20.8* belongs to the small heat-shock protein (sHSP) family and acts as a molecular chaperone to assist correct protein folding and prevent abnormal protein aggregation [64]. The small heat shock protein *BmHsp20.8* during ubiquitination showed significant down-regulated expression in both transcriptome and proteome results. The proteome significantly down-regulated several essential proteins of the ERAD process (*EDEM*, *ERmanI*, *PDIs*, *TRAP*, *Sec61*, *TRAM*, *SVIP*). Therefore, we hypothesize that excessive fluoride accumulation in silkworm’s silk glands causes the accumulation of misfolded proteins in the ER, which leads to ER stress and activation of the UPR of the PERK pathway, as well as activation of ERAD.

## 5. Conclusions

In this study, we revealed the molecular toxicological mechanisms of fluoride exposure through transcriptome and proteome analyses of silkworm’s silk glands. We identified 67 DEGs and 671 DEPs by transcriptome and proteome GO and KEGG enrichment analysis. We found that Ribosome biogenesis in eukaryotes, Protein export, and Protein processing in endoplasmic reticulum were significantly disrupted by fluoride exposure. We speculate that the translation and folding of proteins were blocked, affecting the production of functional proteins and eventually blocking the normal development of silk glands in silkworms under fluoride exposure. The present study hypothesized the mechanism of fluoride effects on silk gland development in silkworms at the transcriptional and protein levels, providing further insight into the potential mechanism of fluoride exposure effects on the organism. Further experiments will be performed subsequently to validate the speculations raised in the present study and investigate whether *BmHEATR1*, *BmSPCS2*, *BmSPCS3*, and *BmHsp20.8* are the target genes/proteins of fluoride exposure.

## Data Availability

Data will be made available on request.

## Supplementary Materials

The following supporting information can be downloaded at https://www.mdpi.com/article/10.3390/biology15201813/s1, Figure S1: Transcriptome validation correlation; Figure S2: The quality control assessment of the LC-MS/MS-based proteomics analysis; Table S1: RT-qPCR primer sequences; Table S2: The quality control parameters of the RNA-Seq data; Table S3: The top 15 significantly enriched pathways of GSEA; Table S4: Differentially expressed genes/proteins that overlap at transcriptional and protein levels; Table S5: Comparison of DEGs’ Log2FC of RNA-seq and qPCR in Ribosome biogenesis in eukaryotes; Table S6: Comparison of DEGs’ Log2FC of RNA-seq and qPCR in Protein export; Table S7: Comparison of DEGs’ Log2FC of RNA-seq and qPCR in Protein processing in endoplasmic reticulum.

## Abbreviations

The following abbreviations are used in this manuscript:

BP	Biological Process
CC	Cellular Component
DEGs	Differentially Expressed Genes
DEPs	Differentially Expressed Proteins
ER	Endoplasmic reticulum
GO	Gene Ontology
GSEA	Gene Set Enrichment Analysis
KEGG	Kyoto Encyclopedia of Genes and Genomes
MF	Molecular Function
SPC	Signal peptidase complex
