# Supplementary material for: Transcriptome and Proteome Changes in Silk Gland of Silkworm (Bombyx mori) Following Oral Feeding of Sodium Fluoride
Source: Biology (Basel). 2026 Oct 10;15(20):1813. doi: 10.3390/biology15201813 (PMC13701516; doi:10.3390/biology15201813)
Supplement: Supplementary file 1 [file biology-15-01813-s001.zip › Figure S.pdf]

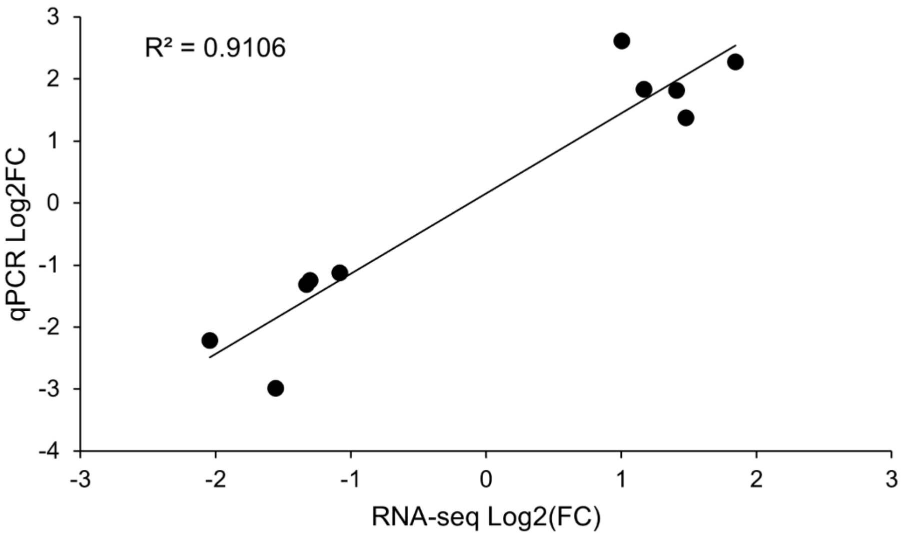

Figure S1. Transcriptome validation correlation. Pearson correlation analysis of $\log_2$(fold change) values between RNA-seq (x-axis) and RT-qPCR (y-axis) for 10 randomly selected differentially expressed genes (200 mg/L NaF vs. control). The coefficient of determination (R^2) is 0.9106, demonstrating high consistency and reliability between the two quantification methods. All experiments were performed with three biological replicates (n = 3).

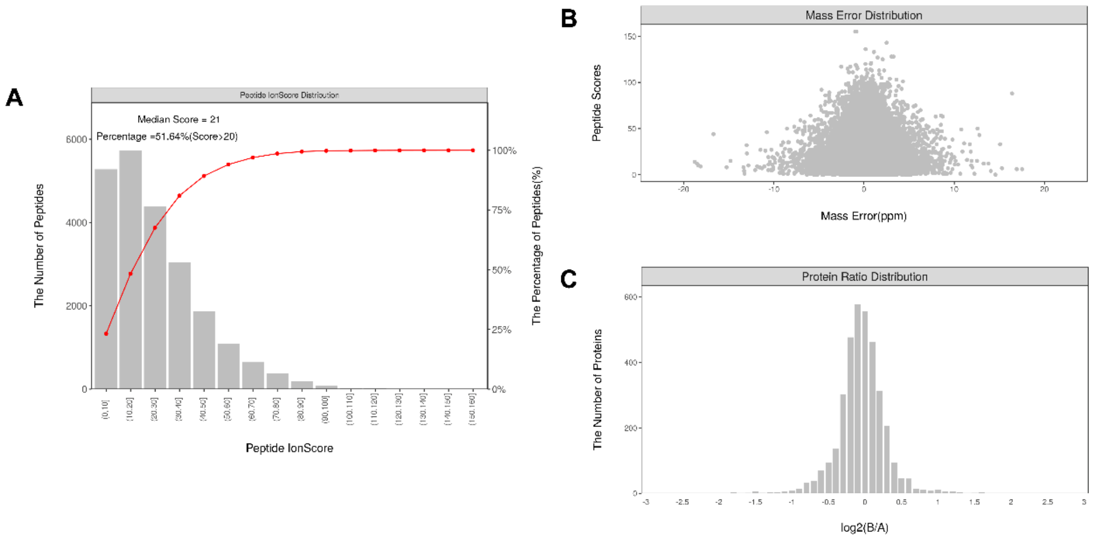

Figure S2. The quality control assessment of the LC-MS/MS-based proteomics analysis. (A) Distribution of peptide ion scores. The MS2 MASCOT scores are of favorable quality: 51.64% of the identified peptides have an ion score above 20, with a median peptide ion score of 21. (B) Mass error distribution of identified peptide ions. The mass deviations of all identified peptides are mainly distributed within 10 ppm, demonstrating high accuracy and reliability of peptide identification. (C) Distribution of protein abundance ratios (NaF treatment vs. control group). The abundance ratios of most proteins between the two sample groups are close to 1, indicating proper data normalization and robust quantitative performance.
