# Supplementary material for: Transcriptome and Proteome Changes in Silk Gland of Silkworm (Bombyx mori) Following Oral Feeding of Sodium Fluoride
Source: Biology (Basel). 2026 Oct 10;15(20):1813. doi: 10.3390/biology15201813 (PMC13701516; doi:10.3390/biology15201813)
Supplement: Supplementary file 1 [file biology-15-01813-s001.zip › Table S.pdf]

Table S1 RT-qPCR primer sequences

Gene name	Primer sequence	Product size (bp)
KWMTBOMO02241	F: ACGGCGGAGGCTGCAAAGAT<br>R: GCCAGAGCACAGAGGAACAGTC	149
KWMTBOMO08011	F: ATTTCTGGGACGATGGCAACGG<br>R: GGAGTGTTGACCAAGAGCCTGG	119
KWMTBOMO00375	F: AGCCAATACCGACAACAGGC<br>R: GTTCGTCCGCTACGATGCAC	249
KWMTBOMO01135	F: GCGGAACCATCTACTCCACC<br>R: ATTCTTCAGTAGGGCTGCGG	134
KWMTBOMO01743	F: AGATGTTCTTGGGCGTCGTT<br>R: AGGGCTGTCTGTTGGTTCAG	244
KWMTBOMO02651	F: GATTGCTGCTGGCATGGTC<br>R: GCTGGTTGAGGGGCAACTAC	123
KWMTBOMO05499	F: CTCCTCAACATCTCTCCGCC<br>R: GCGGGCAGAGTGAAGGTATT	83
KWMTBOMO08448	F: GCATGAGGAGAGGTTTTGCG<br>R: AGCACTTTGTCTCCAGGTCG	225
KWMTBOMO09597	F: CACTCCCATCGGTCAAACCA<br>R: GCTGCTATGCAATTCTCGCC	177
KWMTBOMO09900	F: ACCCAAGCGGAGTTGAAGTT<br>R: TTATGGGCAGCTTACGGGAC	150
KWMTBOMO10946	F: GCATCGGCGATCTTAAAGTTAT<br>R: GCTTTGTGGTTGGAGCAGAG	85
KWMTBOMO12094	F: TGGTTGTATGAAAGGACGCAC<br>R: TGCGTGAAGACAACGAACGA	196
BmHEATR1	F: TCCGAGGGTTTTGATTCCCG<br>R: CAGTGCTGTCAGGAAGAGGG	155
BmUTP6	F: AGAAACCCTGATGCTGAGCC<br>R: TTTCTTCGTCAGTCTCCGCC	88
BmNop10	F: AGCGACCATCGACCCATTTG<br>R: TTGTTGTGTAAGCAGCAGGC	127
BmUTP14	F: GCAACGAGCCCACAAACATC<br>R: TGTCGCTCGCTTTATACGCT	203
BmEMG1	F: GCACAACTGGAAAGCGTGAA<br>R: GGCGACAAGAACCAGGATCA	106
BmRan	F: CACAGCCGGACAAGAGAAGT<br>R: ACCTTCACATACACGGACCA	148
BmRio2	F: GAGTTTGCAGACGCGGTAGA<br>R: GATGACGTCGTTGCGTTTGT	114
BmSPCS2	F: ACGGCGGAGGCTGCAAAGAT	149

	R: GCCAGAGCACAGAGGAACAGTC	
BmSPCS3	F: ATTTCTGGGACGATGGCAACGG<br>R: GGAGTGTTGACCAAGAGCCTGG	119
BmSec61β	F: CCTCAATCTCCGAGTGCTACC<br>R: AGTGCTTCGGTTTCTGGCTG	144
BmSec11	F: ATGATGTGCGGCGAATGAAC<br>R: AAAGCTGGCTCCATACTGCC	160
BmBip	F: GGAGTTTGTTCGCGCTAGTT<br>R: ATGCGGTTTCCCTGGTCATT	175
BmSPR68	F: TGGCACAGGCTAAAGAGTCG<br>R: GGTGCGGATTTCAGTTTCGG	250
BmSRPR	F: ATGCTGCTACCTTACTGCCG<br>R: CTTCCTCACTGCTGCTCTCC	99
BmE2J2	F: ACGTCGTTAAAGGGCCGGAGAA<br>R: CCAAGCAGGGTTCCAAGTGTCC	191
BmHsp1	F: CGGTCAAGGCGTCGAACAACA<br>R: GCGGGTGAACTGACGGGAAATG	92
BmHsp19.9	F: GGAGGCAACGAGGAGGAGAACT<br>R: AGGCAGGCGAACACCGTCAT	140
BmHsp20.8	F: CATCTCGTGACCTCGGTTCCAG<br>R: TCTCTTCGTGCTTGCCTTCCA	142
BmSec61B	F: GCCAGTGGTAGTACAGTCCGTC<br>R: TCCTCCAGAACCAGCACCAGTG	87
BmSec61G	F: CGTCGAGCCTGGCAAGCAATT<br>R: TCCGATGGCTGTTGCAATGGC	109
BmSSR3	F: ACCTGGTTGCTGGCTCTTGC<br>R: CCTGGACATTTCACGGGCAACA	99

Table S2 The quality control parameters of the RNA-Seq data

Sample	Raw reads	Raw bases	Clean reads	Clean bases	Total mapped	Error rate (%)	Q20 (%)	Q30 (%)	GC content (%)
Bom_0_1	57,039,020	8,555,853,000	56,966,898	8,460,586,039	33990610(92.068%)	0.0277	96.67	92.06	48.91
Bom_0_2	42,001,072	6,300,160,800	41,966,348	6,241,123,406	27453513(91.736%)	0.0273	96.81	92.32	48.65
Bom_0_3	49,605,278	7,440,791,700	49,557,164	7,368,049,720	12199700(91.010%)	0.0288	96.15	91.15	50.32
Bom_200_1	47,074,486	7,061,172,900	47,046,930	6,994,130,702	16559261(91.243%)	0.0282	96.42	91.63	49.75
Bom_200_2	47,744,316	7,161,647,400	47,709,428	7,082,782,059	15313915(91.214%)	0.0284	96.31	91.52	50.27
Bom_200_3	44,634,128	6,695,119,200	44,604,666	6,639,220,195	21309756(91.348%)	0.028	96.55	91.72	50.09

Table S3 The top 15 significantly enriched pathways of GSEA

Term ID	Description	setSize	ES	NES	P-adj
ko03060	Protein export	22	-0.720397377	-2.062992243	0.04031
ko00600	Sphingolipid metabolism	28	0.618948238	1.831897875	0.04031
ko03050	Proteasome	36	-0.667418289	-2.117353279	0.04031
ko00073	Cutin, suberine and wax biosynthesis	10	-0.832583221	-1.917544122	0.04031
ko00232	Caffeine metabolism	10	-0.82775131	-1.906415621	0.04031
ko04915	Estrogen signaling pathway	45	-0.582215037	-1.918312842	0.04031
ko04612	Antigen processing and presentation	19	-0.74104952	-2.043817125	0.04031
ko00190	Oxidative phosphorylation	90	-0.556272228	-2.110047448	0.04031
ko04146	Peroxisome	75	-0.581272427	-2.123984261	0.04031
ko01130	Biosynthesis of antibiotics	152	-0.470432699	-1.941913979	0.04031
ko01120	Microbial metabolism in diverse environments	128	-0.452897688	-1.815473829	0.04031
ko04141	Protein processing in endoplasmic reticulum	135	-0.609261541	-2.464144266	0.04031
ko01110	Biosynthesis of secondary metabolites	258	-0.366335902	-1.617591654	0.04031
ko03008	Ribosome biogenesis in eukaryotes	70	0.46530340	1.669045870	0.04999
ko00020	Citrate cycle (TCA cycle)	30	-0.58377526	-1.780699848	0.04999

Table S4 Differentially expressed genes/proteins that overlap at transcriptional and protein levels

NR tophit name	Gene id	Regulate	Description in *Bombyx mori*	KEGG pathways
XP_004929914.1	KWMTBOMO00375	down	probable medium-chain specific acyl-CoA dehydrogenase, mitochondrial isoform X1	ko00071, ko00280, ko00410, ko00640, ko01100, ko01110, ko01130, ko01200, ko01212, ko03320
NP_001040409.1	KWMTBOMO01135	up	eukaryotic translation initiation factor 4E-binding protein 2	ko03013, ko04213
XP_037866437.1	KWMTBOMO01743	up	HEAT repeat-containing protein 1	ko03008
XP_004929234.1	KWMTBOMO01844	down	venom dipeptidyl peptidase 4	ko04974
NP_001040214.1	KWMTBOMO02241	down	signal peptidase complex subunit 2	ko03060
NP_001040269.1	KWMTBOMO05178	down	phosphoserine aminotransferase 1	ko00260, ko00680, ko00750, ko01100, ko01120, ko01130, ko01200, ko01230

NP_001104007.1	KWMTBOMO06852	down	cytochrome P450, family 6, subfamily ab, polypeptide 5	-
NP_001091763.1	KWMTBOMO08011	down	signal peptidase complex subunit 3	ko03060
XP_037872304.1	KWMTBOMO09597	down	cytosolic 10-formyltetrahydrofolate dehydrogenase	ko00670
XP_004928272.1	KWMTBOMO10946	up	probable methylmalonate-semialdehyde dehydrogenase [acylating], mitochondrial isoform X1	ko00280, ko00410, ko00562, ko00640, ko01100, ko01200
XP_037875456.1	KWMTBOMO14218	down	ubiquitin-fold modifier-conjugating enzyme 1	-
NP_001091794.1	KWMTBOMO16003	down	heat shock protein hsp20.8	ko04141, ko04213

Table S5 Comparison of DEGs' Log2FC of RNA-seq and qPCR in Ribosome biogenesis in eukaryotes

Gene name	RNA-seq Log2FC	qPCR Log2FC
BmHEATR1	1.16496694	-0.649365318
BmUTP6	0.049267191	2.777791885
BmNop10	0.805691157	1.484736903
BmUTP14	0.107063627	2.973775125
BmEMG1	0.432639644	4.497675838
BmRan	0.074034049	4.393995365
BmRio2	0.160729451	1.593919555

Table S6 Comparison of DEGs' Log2FC of RNA-seq and qPCR in Protein export

Gene name	RNA-seq Log2FC	qPCR Log2FC
BmBip	-0.629890383	-0.151802668
BmSPCS2	-1.05776992	-1.008937314
BmSPCS3	-1.280483674	-1.558377905
BmSec61β	-1.061204251	-0.610756854
BmSec11	-0.879911944	-0.908569876

BmSRP68	-0.407824199	-0.550703457
BmSRPR	-0.740073457	-1.274243681

Table S7 Comparison of DEGs' Log2FC of RNA-seq and qPCR in Protein processing in endoplasmic reticulum

Gene name	RNA-seq Log2FC	qPCR Log2FC
BmE2J2	-1.026173217	-0.692829546
BmHsp1	1.16496694	2.922179715
BmHsp19.9	-2.066235324	-0.51196007
BmHsp20.8	-2.414557359	-1.900119682
BmSec61B	-1.061204251	-2.072086329
BmSec61G	-1.114685559	-1.750418706
BmSSR3	-1.131989131	-1.730087366
